# Supplementary material for: Transfer of extracellular vesicle‐microRNA controls germinal center reaction and antibody production
Source: EMBO Rep. 2020 Feb 19;21(4):e48925. doi: 10.15252/embr.201948925 (PMC7132182; doi:10.15252/embr.201948925)
Supplement: Supplementary file 1 — Appendix [file EMBR-21-e48925-s001.docx]

**Appendix- SUPPLEMENTARY MATERIALS**

**Table of Contents**

**Appendix Table S1. Predicted targets for selected transferred microRNAs obtained from *in silico* analysis**…………………………………………………………………………………………………………………..Page2

**Appendix Table S2. Antibodies and primers**…………………………………………………………………..Page 3


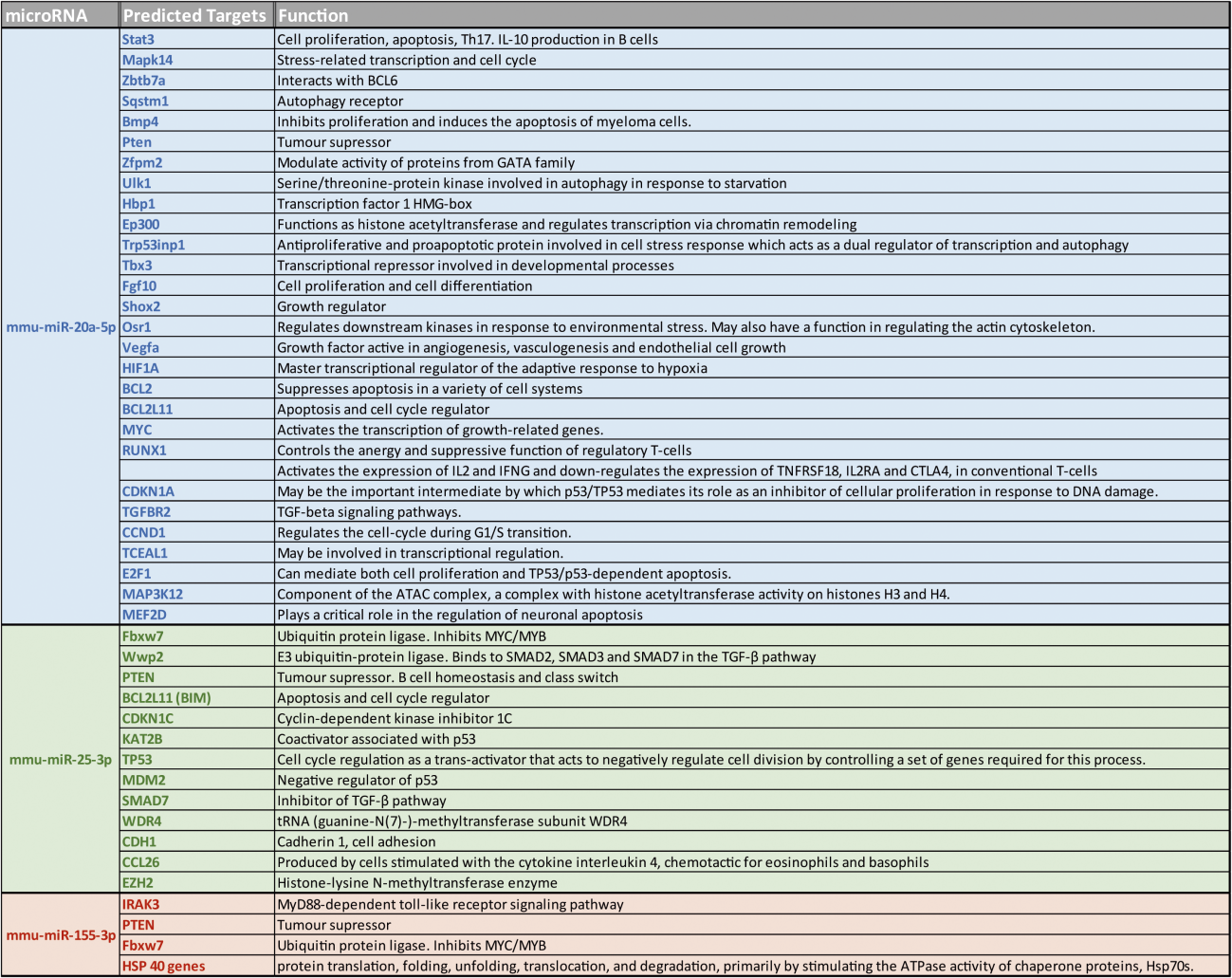


**Appendix Table S1. Predicted targets for selected transferred microRNAs obtained from *in silico* analysis.** The table indicates predicted targets obtained using the miRTARBase and TargetScan algorithms.

| Primers | | |
| --- | --- | --- |
| **Targets** | **Sequence (FOR)** | **Sequence (REV)** |
| **PTEN** | TGGATTCGACTTAGACTTGACCT | GCGGTGTCATAATGTCTCTCAG |
| **Fbxw7** | GTTCCGCTGCCTAATCTTCCT | CCCTTCAGGGATTCTGTGCC |
| **BCL2L11 (BIM)** | GAGATACGGATTGCACAGGAG | CGGAAGATAAAGCGTAACAGTTG |
| **CDKN1C (p57)** | CGAGGAGCAGGACGAGAATC | GAAGAAGTCGTTCGCATTGGC |
| **TP53** | CTCTCCCCCGCAAAAGAAAAA | CGGAACATCTCGAAGCGTTTA |
| **MDM2** | TGTCTGTGTCTACCGAGGGTG | TCCAACGGACTTTAACAACTTCA |
| **Stat3** | CAATACCATTGACCTGCCGAT | GAGCGACTCAAACTGCCCT |
| **CCND1** | GCGTACCCTGACACCAATCTC | CTCCTCTTCGCACTTCTGCTC |
| **GAPDH** | CAT GGC CTT CCG TGT TCC TA | CCT GCT TCA CCA CCT TCT TGA T |
| **DICER** | ACGAAATGCAAGGAATGGACTC | GGCACCAGCAAGAGACTCAAA |

**Appendix Table S2. Antibodies and primers.**
