# Supplementary material for: Transfer of extracellular vesicle‐microRNA controls germinal center reaction and antibody production
Source: EMBO Rep. 2020 Feb 19;21(4):e48925. doi: 10.15252/embr.201948925 (PMC7132182; doi:10.15252/embr.201948925)
Supplement: Supplementary file 2 — Expanded View Figures PDF [file EMBR-21-e48925-s002.pdf]

Expanded View Figures

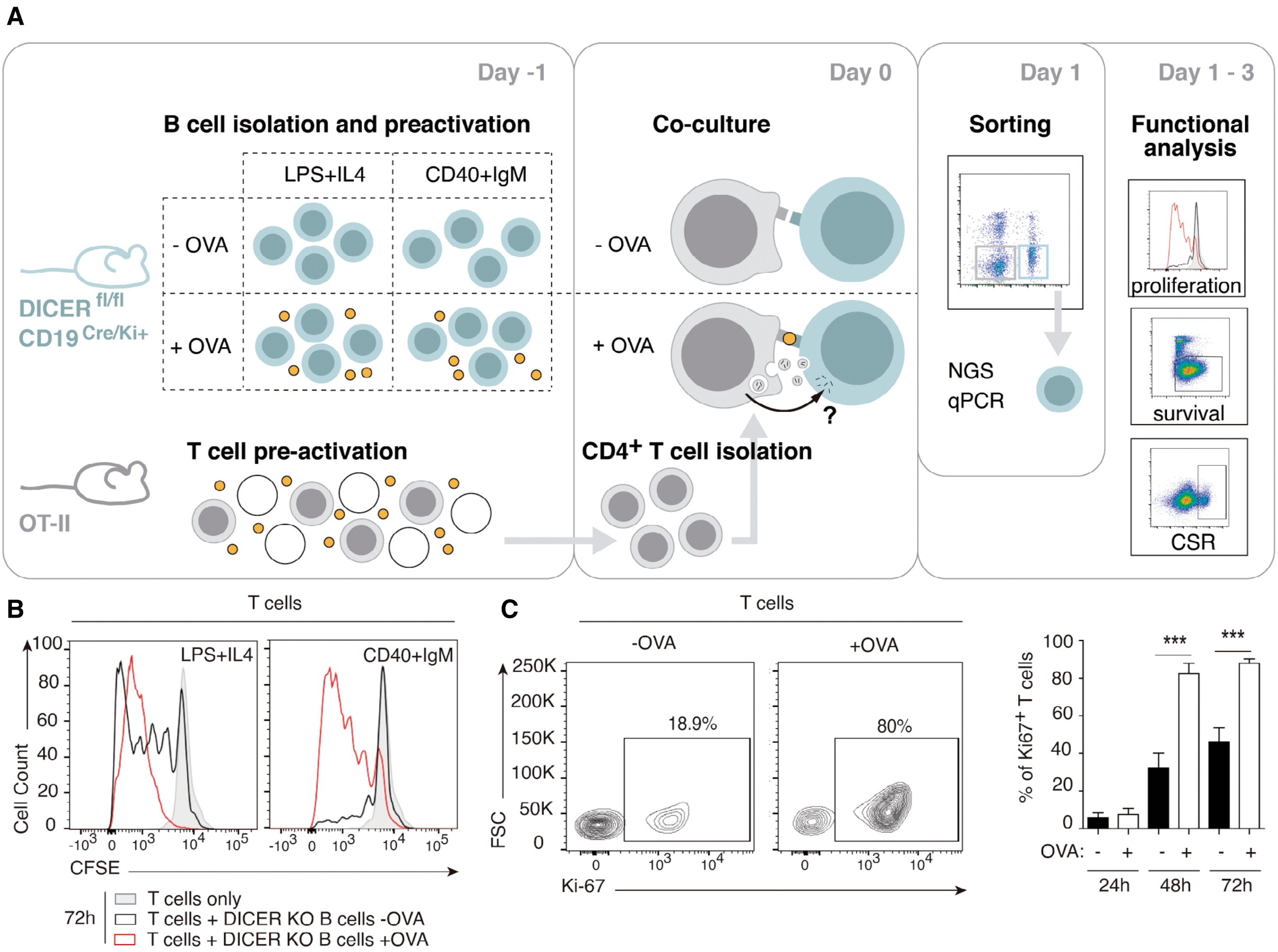

Figure EV1. DICER-KO B cells form mature IS with OT-II-isolated CD4$^+$ T cells in the presence of OVA.

A Model of IS *in vitro* formation between DICER-KO B cells and OTII-derived CD4$^+$ T cells.

B, C Representative flow cytometry analysis of CD4$^+$ T-cell proliferation assessed by Ki67 expression (B) and CFSE labeling of T cells (C) in the presence or absence of OVA after 72 h co-culture with DICER-KO B cells. Bar charts represent the Mean values ± SEM of at least four independent experiments. Significance was assessed by paired Student's *t* test comparing the OVA and NO OVA conditions; $^*P < 0.05$.

© 2020 The Authors

Figure EV2. Transfer of specific miRNAs and regulation of predicted mRNA targets after IS formation.

A Sequence alignment of mouse and human miR-20a-5p, miR-25-3p, and miR-155-3p.

B Quantitative RT–PCR of DICER mRNA levels in our experimental conditions in the absence or presence of OVA. Bar chart shows the mean mRNA levels from $n \geq 3$ independent experiments ± SEM.

C Total counts per million of upregulated microRNAs from small RNA sequencing data. Bar charts show the mean counts per million from three independent experiments. Significance was assessed by comparing *P*-values, which were adjusted with the Benjamini and Hochberg method, and the threshold for changes in small RNA expression was set to adjusted $P < 0.2$; *$P < 0.05$, **$P < 0.01$, ***$P < 0.001$.

D Quantitative RT–PCR showing miRNA levels of DICER-KO CD19Cre$^{Ki/+}$ Dicer$^{fl/fl}$ and DICER-expressing CD19Cre$^{Ki/+}$ Dicer$^{fl/+}$ either untreated or activated for 16 h with a mixture of LPS and IL-4. Data were normalized to RNU1A1 and RNU5G. Bar chart shows the mean mRNA levels from $n \geq 3$ independent experiments ± SEM.

E, F Quantitative RT–PCR of putative selected targets (from *in silico* analyses) after cognate immune interactions between isolated DICER-KO B cells pre-activated with LPS+IL-4 (E) or CD40+IgM (F), and OTII T lymphocytes in the presence or absence of OVA. Bar chart shows the mean mRNA levels from $n \geq 3$ independent experiments ± SEM.

Data information: Significance was assessed by paired Student's *t*-test comparing the OVA and NO OVA conditions; *$P < 0.05$, **$P < 0.01$, ***$P < 0.001$.

© 2020 The Authors

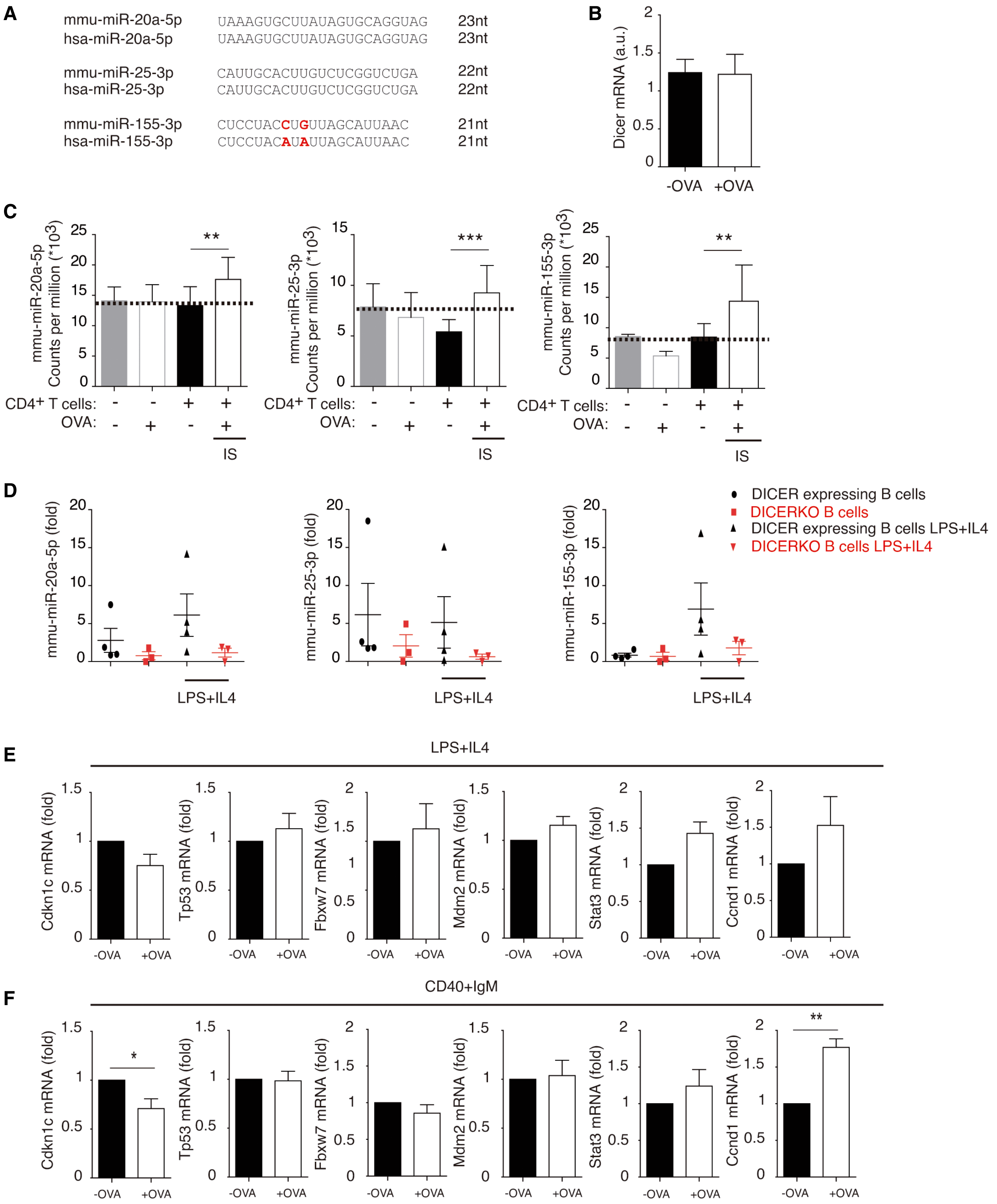

Figure EV2.

 © 2020 The Authors

Figure EV3. Mmu-miR-20a-5p, mmu-miR-25-3p, and mmu-miR-155-3p are detected in OT-II CD4+ T cell-derived EVs.

A Mean counts per million (CPM) analyzed by small RNA NGS of the 6 differentially expressed miRNAs after IS formation in cell and EV fractions of mouse lymphoblast cultures. Bar charts show the mean of three independent experiments ± SEM. Significance was assessed by unpaired Student's *t* test comparing the exosomal and cellular miRNA content; $*P < 0.05$, $**P < 0.01$.

B Schematic representation of the two protocols used for small EV isolation.

C Dot plot of size-exclusion chromatography fractions with anti-CD63 and anti-CD81 antibodies.

D Representation of the different fractions relative expression of CD63 and CD81, assessed by Image Gauge software analysis of dot-plots, and protein concentration measured by Nanodrop. SEC fractions 1, 2, and 3 were pooled and ultracentrifuged at 100,000 *g* for miRNA content analysis by qPCR.

E Quantitative RT–PCR of mmu-miR-20a-5p, mmu-miR-25-3p, and mmu-miR-155-3p expression in SEC fractions and secreting cells, normalized to UniSp6 spike-in. Bar charts show the mean ± SEM of a representative experiment from two independent experiments performed.

F Quantitative RT–PCR showing miRNA levels in medium and OT-II $CD4^+$ T cell-derived small EVs obtained by ultracentrifugation, normalized to UniSp6 spike-in. Bar charts show the mean ± SEM of a representative experiment from two independent experiments performed.

© 2020 The Authors

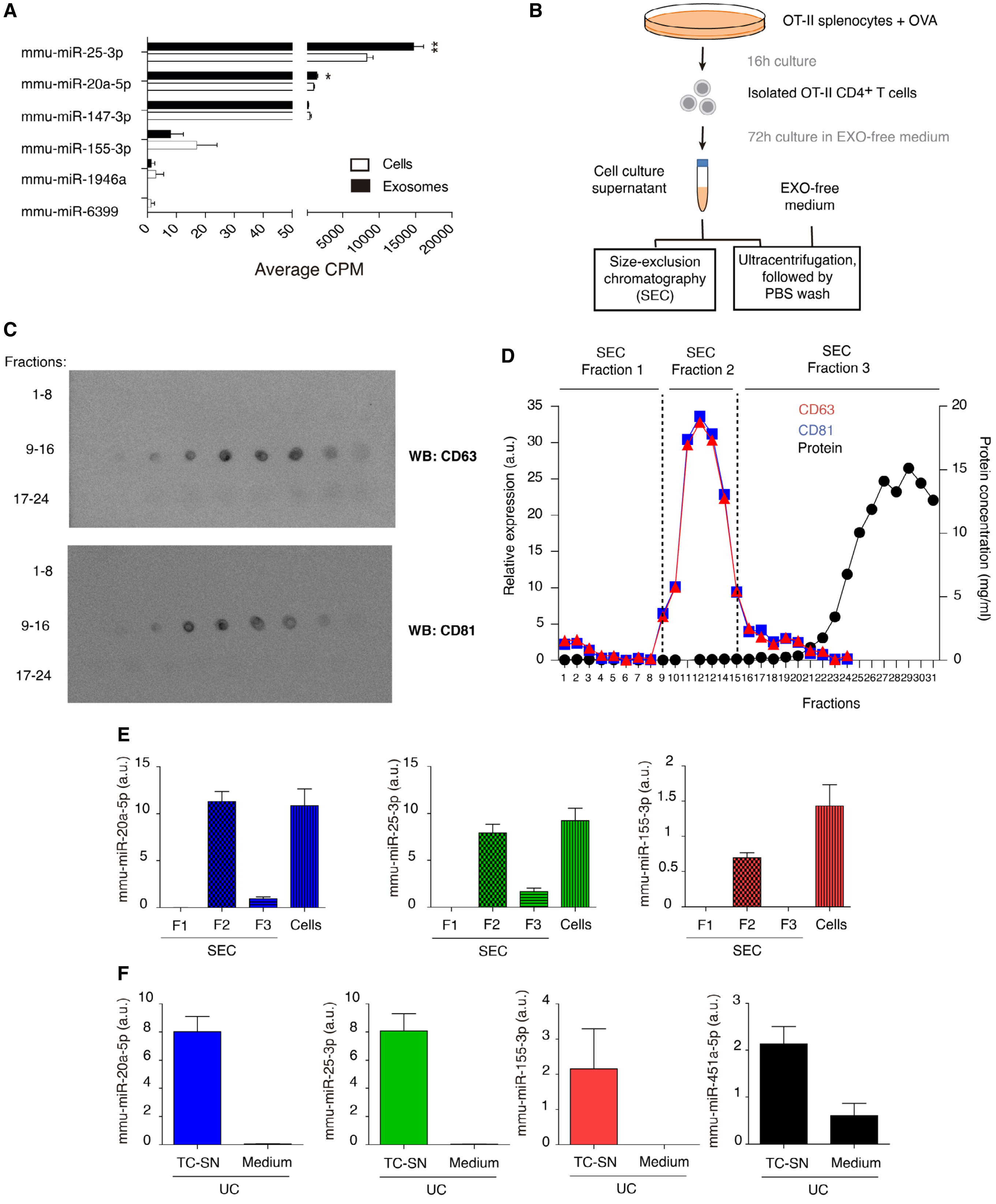

Figure EV3.

 © 2020 The Authors

Figure EV4. Mmu-miR-20a-5p promotes B-cell proliferation and survival *in vitro*.

Fluorescently tagged mimics of the miRNAs mmu-miR-20a-5p, mmu-miR-25-3p, and mmu-miR-155-3p were nucleofected into activated B lymphocytes from C57/BL6 mice.

A–C Lymphocyte proliferation detected by cell violet tracer dilution (A and B) and Ki67 expression (C) 72 h after nucleofection with fluorescently tagged miRNA mimics. Histograms from a representative experiment are shown, and bar charts show the mean percentage of proliferating cells ± SEM. Results are representative of at least 2 independent experiments.

D Dot-plots show survival 48 h after mimic nucleofection from at least 4 independent experiments and bar charts show mean values at 24 h (left) and 48 h (right) after nucleofection, assessed by fixable viability dye staining. Bar charts show the percentage of live B cells ± SEM from at least three independent experiments.

E–G LPS- and IL-4-activated B cells were transduced with precursor mmu-miR-25 retroviral constructs. Bar charts show the percentage of live B cells ± SEM from at least three independent experiments. (E) Quantitative RT–PCR, showing the increase in mature mmu-miR-25-3p in LPS+IL-4-activated B cells after retroviral transduction with precursor mmu-miR-25 retroviral constructs. Expression is normalized to RNU1A1 and RNU5G. (F) Quantitative real-time PCR of the mRNA targets Pten and Bim in pre-miR-25-transfected activated B cells. Expression is normalized to GAPDH. Data are the means of three independent experiments. (G) Density plots of IgG1 expression in pre-miR-25-transfected activated B cells. Plots are representative of at least three independent experiments.

H–J Representative plots showing cell violet dilution (H), Ki67 expression (I), and cell survival analyzed by viability marker and caspase-3 expression (J) after pre-miR-25 B-cell transduction.

Data information: Significance was assessed by paired Student's *t* test comparing the OVA and NO OVA conditions; *$P < 0.05$.

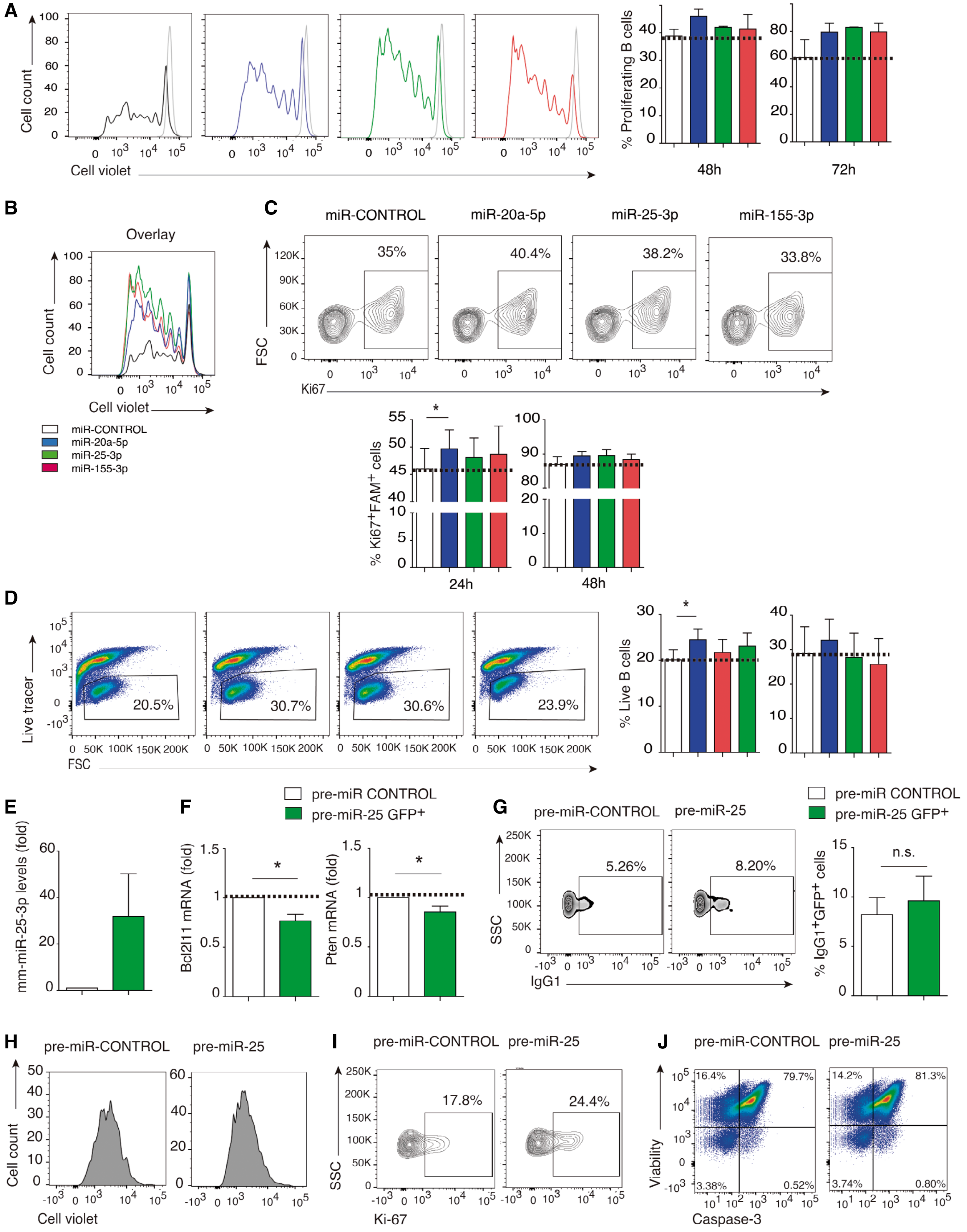

Figure EV4.

© 2020 The Authors

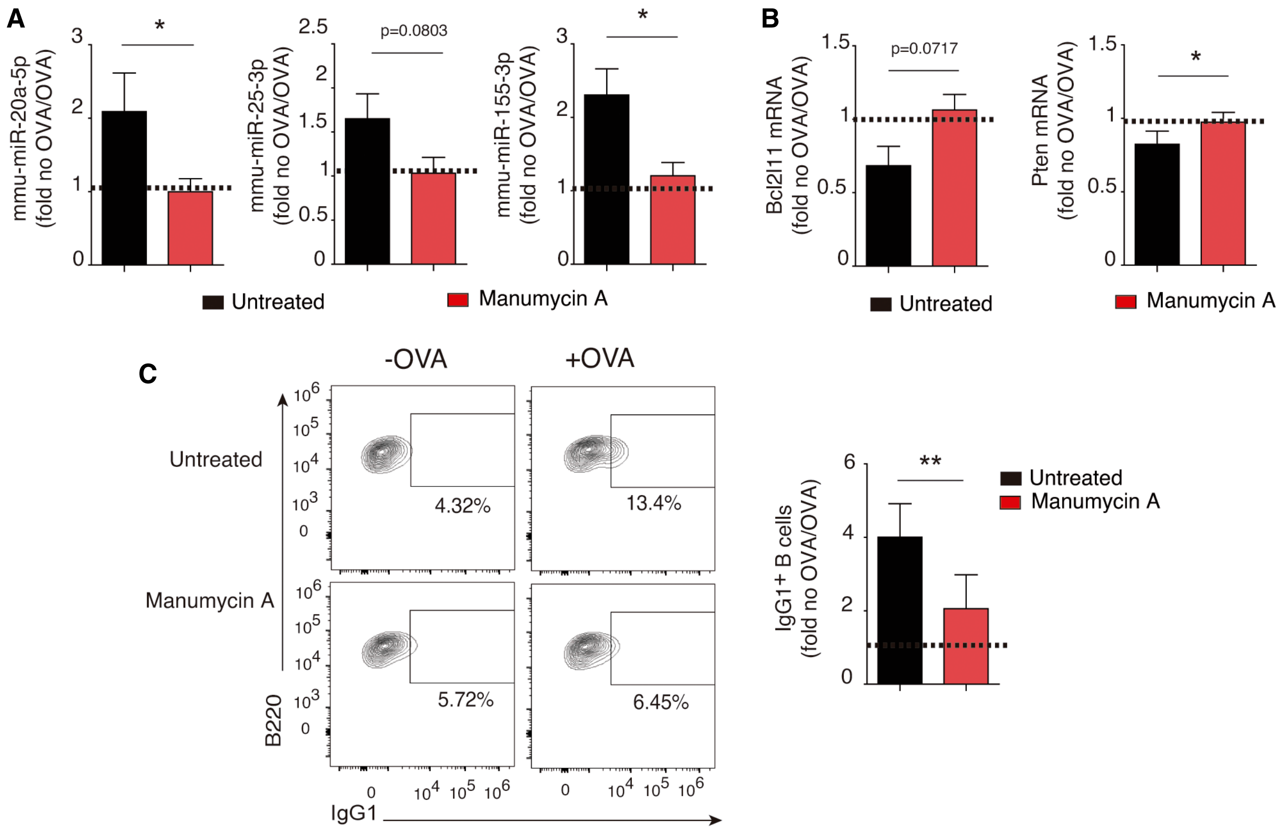

Figure EV5. T-cell EV release is required for B-cell class switching.

Isolated CD4+ T cells were pre-treated with manumycin-A or vehicle before co-culture with isolated DICER-KO B cells.

A Quantitative RT–PCR showing relative expression of mmu-miR-20a-5p, mmu-miR-25-3p, and mmu-miR-155-3p miRNAs after co-culture in the presence or absence of OVA. miRNA expression is normalized to RNU1A1 and RNU5G. Data are expressed as the fold-change in the OVA versus NO OVA conditions, and the mean from at least three independent experiments ± SEM is shown

B Quantitative real-time PCR showing DICER-KO B-cell mRNA target down-modulation after co-culture with control or manumycin-A pre-treated OTII T cells. Expression is normalized to GAPDH. Bar charts show the mean from at least three independent experiments ± SEM.

C Dot-plot analysis of IgG1 switch after manumycin-A-dependent EV release blockade. A representative plot is shown of at least two independent experiments. Bar charts show the mean ± SEM.

Data information: Significance was assessed by paired Student *t* test comparing the OVA and NO OVA conditions; $*P < 0.05$, $**P < 0.01$.

© 2020 The Authors
